# Oligoribonuclease mediates high adaptability of *P. aeruginosa* through metabolic conversion

**DOI:** 10.1186/s12866-023-03175-3

**Published:** 2024-01-19

**Authors:** Lulu Yang, Lili Wang, Mengyu Wang, Ousman Bajinka, Guojun Wu, Ling Qin, Yurong Tan

**Affiliations:** 1 Department of Respiratory Medicine, Xiangya Hospital, Central South University, Changsha, Hunan 410078 China; 2 Department of Medical Microbiology, Xiangya School of Medicine, Central South University, Changsha, Hunan 410078 China

**Keywords:** *P. aeruginosa*, Oligoribonuclease, Pathogenicity, c-di-GMP, Biofilm

## Abstract

**Background:**

Oligoribonuclease (*orn*) of *P. aeruginosa* is a highly conserved exonuclease, which can regulate the global gene expression levels of bacteria through regulation of both the nanoRNA and c-di-GMP. NanoRNA can regulate the expression of the bacterial global genome as a transcription initiator, and c-di-GMP is the most widely second messenger in bacterial cells.

**Objective:**

This study seeks to elucidate on the regulation by *orn* on pathogenicity of *P. aeruginosa*.

**Methods:**

*P. aeruginosa* with *orn* deletion was constructed by suicide plasmid homologous recombination method. The possible regulatory process of *orn* was analyzed by TMT quantitative labeling proteomics. Then experiments were conducted to verify the changes of Δorn on bacterial motility, virulence and biofilm formation. Bacterial pathogenicity was further detected in cell and animal skin trauma models. ELISA detection c-di-GMP concentration and colony aggregation and biofilm formation were observed by scanning electron microscope.

**Results:**

*orn* deletion changed the global metabolism of *P. aeruginosa* and reduced intracellular energy metabolism. It leads to the disorder of the quorum sensing system, the reduction of bacterial motility and virulence factors pyocyanin and rhamnolipids. But, *orn* deletion enhanced pathogenicity in vitro and in vivo, a high level of c-di-GMP and biofilm development of *P. aeruginosa*.

**Conclusion:**

*orn* regulates the ability of *P. aeruginosa* to adapt to the external environment.

**Supplementary Information:**

The online version contains supplementary material available at 10.1186/s12866-023-03175-3.

## Introduction

*P. aeruginosa* is a common gram-negative opportunistic pathogen isolated in hospitals and other health posts. This bacteria can colonize almost all over the body, such as respiratory tract, vascular endothelium, skin and soft tissue, digestive tract, urinary system and central nervous system, causing a health burden of nosocomial infections [1, 2]. These opportunistic pathogens are specifically susceptible to immunodeficiency, burns, cystic fibrosis and other diseases, and have a high incidence rate and mortality [3]. *P. aeruginosa* has many virulence components, including bacterial structural components, such as lipopolysaccharide, flagella and pili; extracellular secretory virulence factors, such as protease and pyoverdine, and a variety of virulence secretion systems, including type III secretion system related to acute infection [4]. Even more adverse mechanism is that *P. aeruginosa* has natural resistance to a variety of antibiotics. This resistance is largely due to the multiple drug efflux system encoded on the chromosome and the low permeability of cell membrane, which makes it difficult for drugs to enter [5, 6]. In addition, biofilm is also the reason why *P. aeruginosa* is difficult to be completely removed. It contains a variety of biological macromolecular substances such as proteins, polysaccharides, nucleic acids, peptidoglycans, lipids and phosphoric acids, as well as macromolecular polymers secreted by bacteria, metabolites and bacterial lysates [7]. Morever, it poses challenges to be cleared by the immune system, which is the main reason for the continuous infection of bacteria [8, 9]. Clinically, the treatment of medical material-related infection caused by biofilm is more difficult, and it is believed that chronic refractory lower respiratory tract infection caused by *P. aeruginosa* is also closely related to biofilm formation [10]. *P. aeruginosa* is sensitive to environment changes and can adapt environment quickly. Meanwhile it has complex virulence system. This subsequently leads to high morbidity and mortality [11]. Therefore, in-depth study of its global regulation mechanism can bring new theoretical basis for the prevention and treatment of clinical *P. aeruginosa* infection.

The oligoribonuclease (*orn*) of *P. aeruginosa* is a highly conserved 3′-5′ exonuclease [12]. It was first found in *E. coli* that can specifically degrade 2 -5nt mRNA, named oligonucleotide exonuclease [13]. In *P. aeruginosa*, *orn* is essential for the hydrolysis of pGpG. The normal degradation of pGpG is crucial for the metabolic cycle of the second messenger c-di-GMP in bacteria. C-di-GMP is synthesized by diguanylate cyclases (DGCs) containing GGDEF domain and degraded by phosphodiesterases (PDEs) with EAL or HD-GYP domain. The PDEs will degrade c-di-GMP to pGpG, and finally degraded by ORN [14–16]. C-di-GMP can dynamically regulate the metabolism of *P. aeruginosa*. High concentration of c-di-GMP can promote the formation of biofilm and reduce bacterial toxicity and movement. These occur mainly during chronic infection, resulting in continuous and reoccuring infections in the host. Low concentration of c-di-GMP will promote bacterial virulence and movement, leading to acute infection when the host immunity decreases [17, 18]. Previous studies have shown that the accumulation of pGpG will inhibit the enzyme function containing EAL domain, increase the level of c-di-GMP in bacteria, enhance the ability of bacterial biofilm formation, and reduce the sensitivity of bacteria to antibiotics [19]. In addition, *orn* is also essential for the degradation of nanoRNA. NanoRNA is a short chain nucleotide in the process of mRNA degradation. It can play a role as a transcription initiation primer, regulate the global gene expression level in bacteria, change the intracellular metabolic pathway, and systematically adjust bacterial activities to adapt to the environment [20]. The exonuclease encoded by *orn* is different from the previously found, and the deletion will lead to the elimination of *E. coli* [13, 21]. In *Bacillus subtilis*, there is no protein homologous to *orn*, but there are many proteins with similar functions, such as Ytql [22, 23]. At present, there are few studies on *orn*, mainly focusing on the effect of *orn* on c-di-GMP metabolism. Jin has been focusing on the research of Orn on virulence factors and drug resistance of *P. aeruginosa* and found that *orn* is essential for the normal expression of type III secretory system (T3SS) gene and the synthesis of pyocyanin [24, 25]. Meanwhile, *orn* mutant strain is sensitive to aminoglycosides β-lactams and ciprofloxacin [26]. The above shows that in-depth study of *orn* gene function is also crucial for exploring the pathogenicity and drug resistance of *P. aeruginosa*. More importantly, in *P. aeruginosa*, *orn* can still survive after knockout, which makes it possible to study the function of *orn* in pathogenic bacteria.

*P. aeruginosa* genome is huge, about 6.2 Mb, and there are still many gene functions to be studied. The life process regulatory network involves a variety of signal pathways to synthesize proteins for life activities [27]. Therefore, perhaps RNA-seq technology alone can’t accurately reflect the gene expression in the life process of bacteria, because gene transcription and translation are ultimately attributed to proteins. Based on the above, we used quantitative proteomics to study the regulation of *orn* deletion on the pathogenicity of *P. aeruginosa*, including virulence factors, bacterial motility and biofilm formation, and verified it at the cellular and animal levels.

## Materials and methods

### Bacterial strains and cells culture

Bacterial strains, and cells used in this study were listed in supplementary Table S1. *P. aeruginosa* ATCC 27853 was grown at 37 °C in Luriab Bertani broth (LB, Sangon Biotech) liquid medium or LB agar medium. A549 cells (ATCC, USA) were cultured in F-12 K medium with 10% fetal bovine serum (FBS).

### Construction of *orn* deletion strain in *P. aeruginosa*

The strains, plasmids and primers used in the experiment were shown in supplementary Table S1 and S2. The upstream and downstream homologous recombination arms of *orn* were amplified from the genome of *P. aeruginosa* ATCC 27853; gentamicin resistance gene (*Gm*) was amplified from pJQ200SK plasmid. The upstream and downstream homologous recombinant arms and *Gm* were connected by overlap PCR to obtain a complete target fragment Δorn:*Gm* (upstream homologous arm-Gm-downstream homologous arm). Then it was cloned into suicide plasmid pCVD442 to construct targeting plasmid pCVD442-Δorn:*Gm*. Next, the target plasmid was transformed into *E. coli* β2155 by electrotransformation, which was called donor bacteria *E.coli* β2155/pCVD442Δorn:*Gm*. Subsequently, the donor bacteria were conjugated with *P. aeruginosa*, and the gentamicin resistant *P. aeruginosa* colonies were screened on the gentamicin plate with a concentration of 33 µl/ml, indicating that the genome of *P. aeruginosa* was integrated with a targeting plasmid, named as ATCC 27853/pCVD442-Δorn:*Gm*. Finally, ATCC 27853/pCVD442-Δorn:*Gm* bacterial fluid was coated on LB plate containing 10% sucrose and cultured until monoclonal was formed. This monoclonal strain was the clone whose *orn* gene was replaced by *Gm*, named ATCC 27853/ Δ orn:*Gm* (Δorn).

### TMT labeled quantitative proteomic analysis

The bacterial suspension cultured overnight was diluted with LB medium to OD600 equal to 0.1, then cultured overnight again. The next day, the bacteria were collected after centrifugation, and then 4 times the volume of lysis buffer containing urea (8 M) and 1% protease inhibitor were added, ultrasonic lysed, and the protein concentrations of the supernatant were measured. The same amount of protein from each sample was taken for enzymatic hydrolysis. Slowly, 20% trichloroacetic acid (TCA) were added, mixed by vortex and precipitated at 4 ℃ for 2 h. After centrifugation, the precipitate was washed with precooled acetone for 2–3 times. Then the precipitate was dried, and trypsin in the ratio of 1:50 (protease: protein, m /m) was added and placed overnight. Dithiothreitol (DTT) was added with the final concentration of 5 mm and reduced at 56 ℃ for 30 min, alkylated with iodoacetamide for 15 min at room temperature in the darkness, diluted with 100 M triethyl ammonium bicarbonate (TEAB) and labelled according to the operating instructions of the TMT kit. The peptide were graded by high pH reversed-phase HPLC with Agilent 300 extend C18 columm (5 µM particles, 4.6 mm inner diameter, 250 mm length). Briefly, the peptides were first separated with a gradient of 8-32% acetonitrile (pH 9) into 60 components, then the peptide segments were combined into 14 components and dried by vacuum centrifuging. The labeled peptides were separated by liquid chromatography-mass spectrometry (LC-MS/MS). MS/MS data containing all the peptides information were identified and analyzed by software Maxquant (v1.6.15.0) using the Uniport-GOA database (http://www.ebi.ac.uk/GOA/), InterProScan (http://www.ebi.ac.uk/interpro/), and Gene Ontology(GO) annotation (http://geneonfelogy.org/). All identified proteins were divided into three categories (cellular components, molecular functions and biological processes) by GO analysis. In order to confer the significance of the differences, the relative quantitative value of each protein in two comparison samples was tested by *t*-test, and the default *p* ≤ 0.05. Prior to the test, the relative quantitative value of protein needs log2 logarithmic conversion. Through the above differential analysis, when *p* value ≤ 0.05, the threshold change of differential expression exceeding 1.3 was taken as significance for up-regulation, and less than 1/1.3 was deemed for down regulation.

### Bacterial growth curve and motility test

Overnight cultured bacterial suspension was diluted to OD600 at 0.1, then it was constantly shaken and the bacterial suspension was taken out every 2 h to detect the absorbance at OD600 for bacterial growth curve. The initial concentration of bacterial suspension was adjusted to OD600 equal to 1. For swarming and swimming assay, 5ul of bacterial suspension were added on the surface of solid medium. Swarming medium included 0.5% agar, 5 g/L glucose, 10 g/L tryptone, 5 g/L yeast extract power, 10 g/L NaCl and swimming medium included 0.3% agar, 10 g/L tryptone and 10 g /L NaCl. For twitching assay, 5 ul of bacterial suspension were punctured into the culture medium including 1% agar, 5 g/L glucose, 10 g/L tryptone, 5 g/L yeast extract power, 10 g/L NaCl. The plates were placed in a constant temperature incubator at 37 °C.

### Virulence factor detection

Pyocyanin is a protein complex released by *P. aeruginosa* to kill bacteria that compete with it for resources.Pyocyanin is a kind of phenazine compound, which is not only a virulence factor, but also a quorum sensing signal molecule to coordinate the changes of *P. aeruginosa* to the environment [28]. Overnight cultured bacterial suspension was diluted to OD600 at 0.1 and shaken or inoculated in a 12 well plate for static culture. The supernatant was collected after culture for 8 h, 1 d, 2 d and 3 d for pyocyanin detection. Briefly, after centrifugation, 1/2 volume of chloroform was added to the supernatant for extraction. Then 1 ml of 0.2 M of HCl was added to the lower organic layer, vibrated fully and stood still. After centrifugation, the upper was taken, and measured at OD520. The detected value multiplied by 17.072 was the final concentration of pyocyanin, that is, the concentration of pyocyanin (ug/ml)=A520 × 17.07. For rhamnolipids detection, after shaking culture for 12 h, 200 ul of bacterial suspension was collected and 1.8 ml of orcinol was added evenly. The bacterial suspension was then boiled in boiling water for 15 min and measured at OD520. The rhamnolipids production was calculated according to the rhamnolipids standard curve.

### Detection of bacterial virulence with A549 cells

In all experiments, cells with culture medium were blank group (Blank), cells with culture medium containing *P. aeruginosa* ATCC 27853 were control group, finally cells with culture medium containing *P. aeruginosa* ATCC 27853 with *orn* deletion were the experimental group (Δorn). Cells were cultured in Ham’s F-12 K (F12K, Solarbio) medium supplemented with 10% fetal bovine serum (FBS, BI) at 37 °C with 5% CO_2_. Bacteria were co-cultured with A549 cells monolayers with MOI 40 for 1–6 h. Subsequently, A549 cells were processed and assayed for cell cytotoxicity and bacterial attachment. Briefly, after cells and bacteria were co-cultured for 4 h, the cells were washed by PBS and then resuspended in 100 ul of binding buffer and icily incubated with 5 µl of annexin V-FITC and 10 µl of propidium Iodide (PI) for 15 min in dark. Then the samples were diluted with 200 ul of binding buffer and loaded to a DxP Athena™ flow cytometer (China) within 1 h. For analysis of bacterial attachment, after 4 h of co-cultured, non-adherent bacteria in the supernatant were aspirated and quantified by serial dilutions as CFU/ml.

### Detection of bacterial virulence in vivo

SD-grade male rats (Hunan SJA Laboratory Animal CO., LTD, *n* = 4) of 8 weeks old weighing about 350 g were used in this experiment. Rats were anesthetized and the dorsal trunk was shaved and surgically prepared. Along the midline of the rat’s back, the skins were marked with a biopsy sampler (d = 8 mm), and then cut off with scissors. The three wounds both on the left and right, each wound on the same side was 2 centimeter away, with the order normal control group, PBS group and Δorn group. Then, the wound was closed with medical tape, and 50 ul bacterial suspension (1 × 10^8^ CFU/mL) was injected under the tape. Same amount of PBS was as blank control. After three days of infection, full-thickness wound-tissue were collected for microbiological and pathological analysis. Biopsies were weighed, placed in 1 ml of PBS and full grounded. The resulting solutions were serially diluted and plated on *P. aeruginosa* screening medium CN agar (Hopebio, China) and incubated at 37 °C overnight. CFU were calculated per gram of tissues. For pathological analysis, the slides were stained with Hematoxylin solution for 3–5 min, followed by rinsing with tap water. Then the slides were treated with Hematoxylin Differentiation solution and rinsed with tap water. Then after treating the section with Hematoxylin Scott Tap Bluing and rinsing with tap water, the slides were soaked in 85% ethanol for 5 min, 95% ethanol for 5 min, and finally in eosin dye for 5 min. After dehydration, the slides were observe with microscope for image acquisition and analysis.

### Cyclic diguanylate GMP(c-di-GMP) detection

Overnight cultured bacterial liquids were diluted to OD600 equal to 0.1. After shaking or static culture for 12 h, the bacterial suspension was taken out for detection. At the same time, for detecting the change of c-di-GMP in the process of biofilm formation, the bacterial suspension also were kept still for 1, 3 and 6 days. The concentration of c-di-GMP were detected by c-di-GMP ELISA kit (Fan-kel, China).

### Biofilm formation assay

Overnight cultured bacterial suspension was diluted to OD600 equal to 0.1, then put into a silicone tube (0.6 mm diameter, 2 cm length) for continuous culture without changing the culture medium. The silicone tube was taken out on the first, third and sixth days of culture for crystal violet staining. Briefly, the tube was softly washed using PBS, and then placed in the drying oven at 60℃ for 30 min. Then the biofilm were dyed with 1% crystal violet for 30 min, gently rinsed with clean water to remove the unbound crystal violet. And finally 1 ml of 33% glacial acetic acid was added to dissolve it for 20 min followed by vortex oscillation for 5 min. The absorbance at OD560 were measured. Four parallel tubes were set in each group and the experiment was repeated for the third time.

### Bacterial viability test

Overnight cultured bacterial suspension s were diluted to OD600 equal to 0.1, then put into a silicone tube (0.6 mm diameter, 2 cm length) for continuous culture without changing the culture medium. The silicone tube was taken out on the first, third and sixth days of culture for viability test. For the bacterial suspension, 100 ul bacterial suspension was absorbed and add 10 ul Alamar Blue detection reagent (Solarbio,China). After incubation in a 37 °C incubator for 4 h without light, the fluorescence enzyme labeling instrument was used for detection. The excitation light wavelength was 560 mm and the emission light wavelength was 590 mm. The relative fluorescence unit (RFU) was recorded. For the bacteria in the biofilm, gently clamp out the silicone tube with tweezers, gently rinse it with PBS for three times, add 1 ml PBS solution and shake the vortex for 5 min to make the bacteria adsorbed on the silicone tube fall off, obtain the biofilm bacterial suspension and detect the bacterial activity,The blank control group used LB medium, all of which were four multiple wells.

### Scanning electron microscopy (SEM)

In order to observe the impact of *orn* on the morphology and structure of *P. aeruginosa*, the overnight cultured bacterial suspension was diluted to OD600 equal to 0.1. Sterile round overslip were put and 2 ml bacterial suspensions were added into a 6-well culture plate. After 8 h to 6 d of culture, round coverslip were washed with PBS and fixed overnight in 2.5% glutaraldehyde at 4 ℃.The next day, the samples were dehydrated by sequentially immersing in 30%, 50%, 70% and 100% propanol solutions for 20 min respectively and then air dried and coated with platinum vapor (about 30 nm thickness) and observed by scanning laser electron microscope (Hitachi S-3400 N).

### RNA extraction and quantitative real time PCR

The bacterial suspension cultured overnight was diluted with LB medium to OD600 equal to 0.1 and cultured overnight again. The bacterial RNA was extracted with RNAprep Pure Cell/Bacteria Kit (TIANGEN, China), then cDNA was transcribed using PrimeScript™ RT Master Mix (taraka). Ultimately, RT-qPCR was performed with SYBR premix extaq II (Takara) with *rpoD* as the housekeeping gene, and the primers were listed in supplementary information supplementary Table S2. Use ΔΔ CT method (Fold Chang = 2^− ΔΔ CT^) calculate the relative expression of genes.

## Results

### *Orn* regulated metabolism of *P. aeruginosa*

**Fig. 1:**
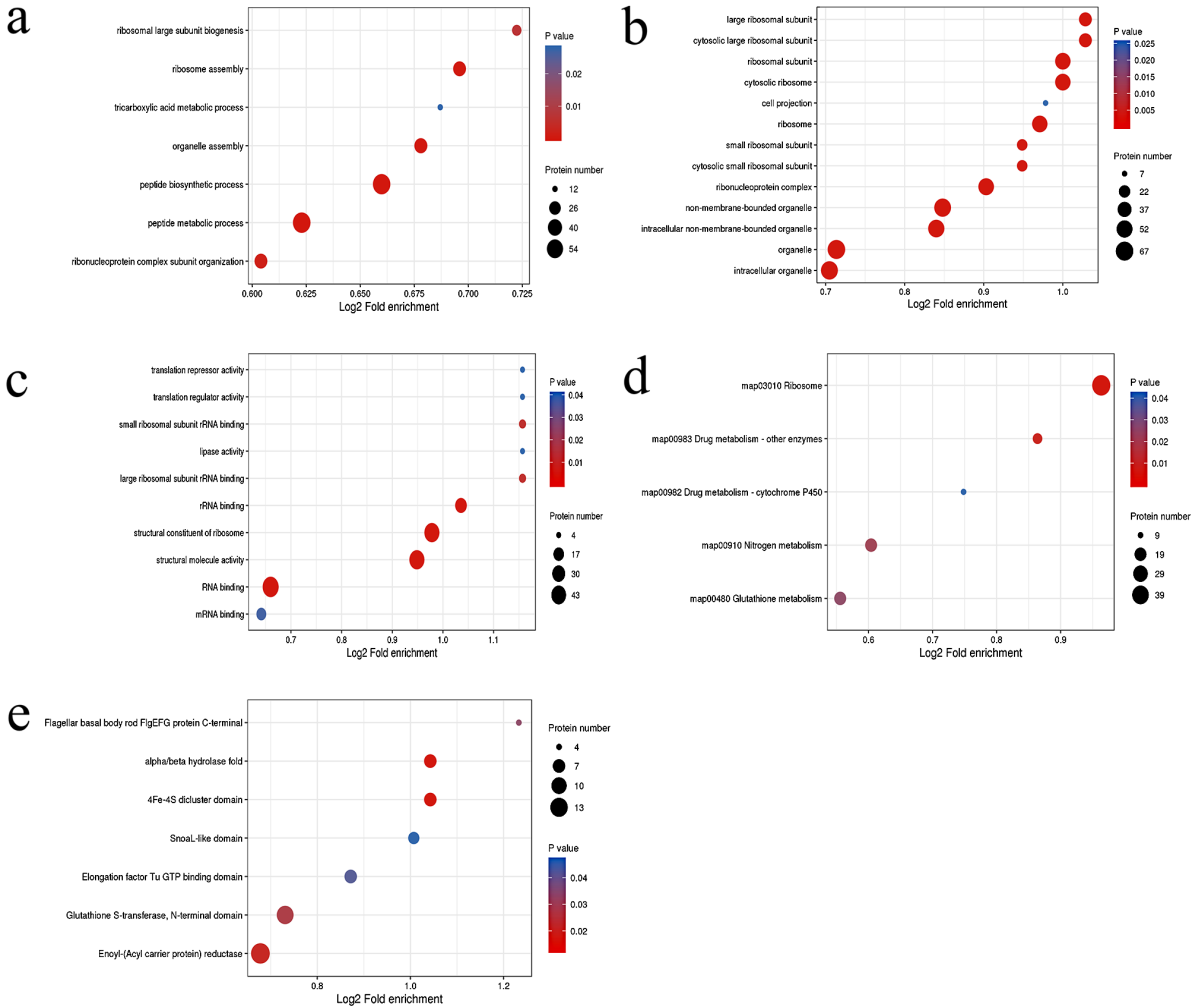
GO enriches bubble map, including biological process, cellular component and molecular function. The vertical axis is each classification under each level 1 classification of GO, and the horizontal axis is the value of fold enrichment after log2 conversion. In the figure, the circular area represents the number of differential proteins, and the circular color represents the enrichment significance p value of differential proteins under go classification. The more red and right the circle is, the more important the classification is

We constructed *orn* deletion strains and used whole protein TMT labeling quantitative proteomics to explore the function of *orn*. 33,340 unique peptides and 4125 proteins were identified. Based on the 1.3-fold difference of expression and the adjusted *p* value less than 0.005, 1058 proteins were found to have decreased expression and 732 proteins had increased expression (Fig. S1a–c). GO analysis was used to identify the details and centralized enrichment pathways of these differentially expressed proteins from protein cellular components, molecular functions and biological processes. The increased expression proteins were mainly concentrated in the ribosomal subunit, cytosolic ribosome, ribosome, structural constituent of ribosome, structural molecular activity, peptide biosynthesis process and peptide metabolic process. Down regulated proteins mainly focused on oxidoreductase activity, plasma membrane succinate dehydrogenase complex, carboxylic acid catabolic process, tricarboxylic acid metabolic process and respiration (Fig. 1). KEGG pathway enrichment analysis showed that the most significantly differentially expressed proteins were concentrated in ribosome, drug metabolism, nitrogen metabolism and cytochrome P450 (Fig. S1d).

Subsequently, we selected several pathways related to the basic survival of *P. aeruginosa* for analysis. Citrate cycle (TCA cycle), fatty acid metabolism, bacterial chemotaxis, quorum sensing, two-component system, glutathione metabolism and flagellum assembly were decreased in varying degrees. Moreover, the beta-lactam resistance pathway was significantly up-regulated (Fig. S2). In general, *orn* deletion significantly affects the transcription and translation process of *P. aeruginosa*. At the same time, bacterial metabolism becomes slow, which may lead to insensitivity to changes in living environment and adverse to the growth of *P. aeruginosa.*

### *Orn* inhibited the growth and movement ability of *P. aeruginosa*

**Fig. 2:**
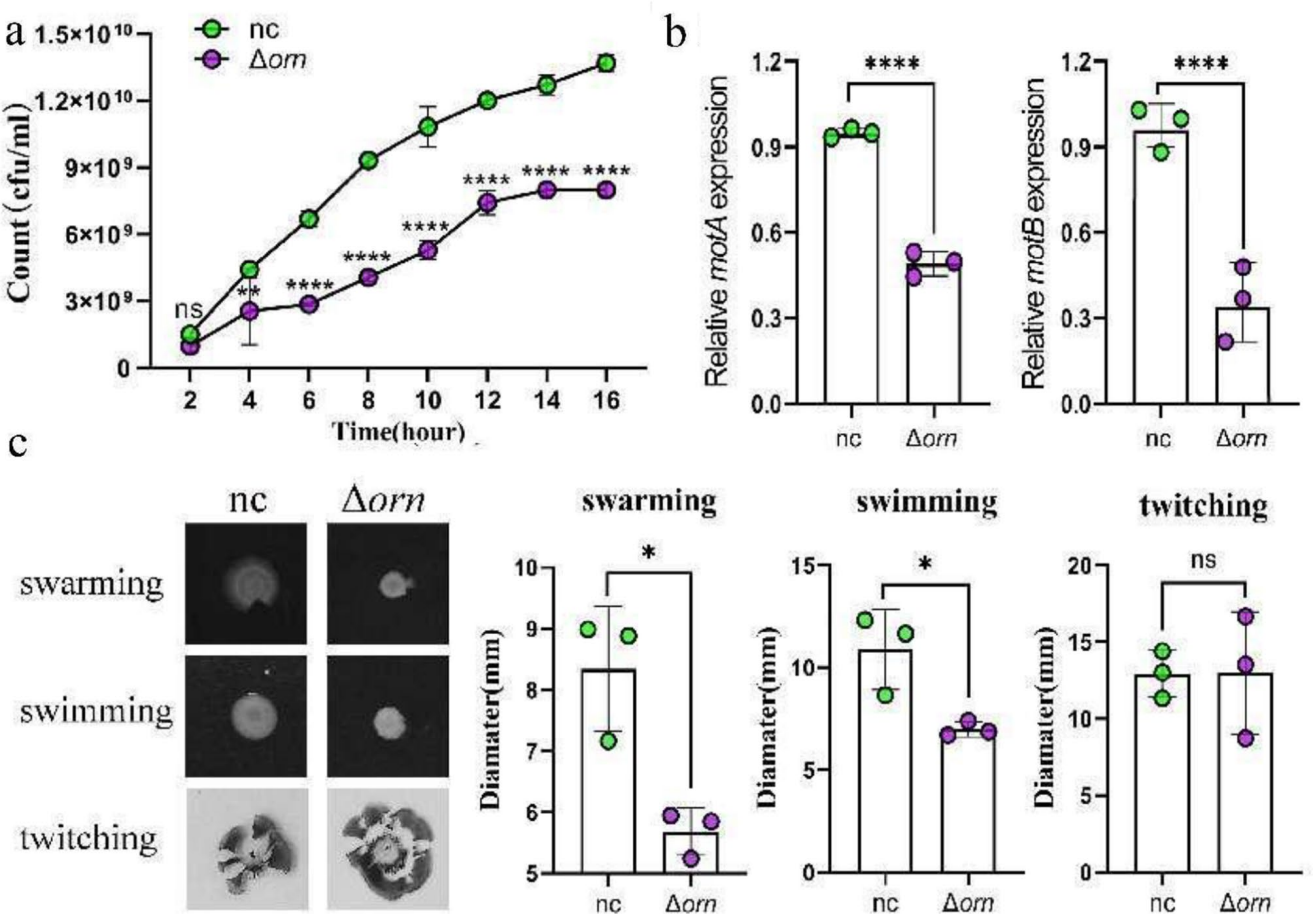
*orn* deletion can reduce the motility of *P. aeruginosa* (*n* = 4). (**a**) Growth curve of *P. aeruginosa* within 16 h. Significance tested with two-sided student’s *t* test, followed by multiple comparison using the Sidak’s test. ***p < 0.001, ****p < 0.0001. (**b**) The relative expression levels of *motA* and *motB* in *P. aeruginosa* was determined by qRT-PCR with *rpoD* as the control housekeeping gene. Significance tested with two-sided student’s *t* test. ****p < 0.0001. (**c**) The motilities of swimming, swarming, and twitching of *P. aeruginosa* in the presence or absence of *orn*. Image J was used to detect three kinds of moving area. Significance tested with two-sided student’s *t* test. **** *p* < 0.0001, * *p* < 0.05

In order to verify the effect of *orn* on the growth of *P. aeruginosa*, we detected the bacterial growth curves of the two strains respectively. We found that the growth of Δorn was significantly slower than that of the normal control group, and its growth rate was significantly inhibited, indicating that *orn* is very important in regulating the proliferation and growth of *P. aeruginosa* (Fig. 2a). We further examined the effect of *orn* on bacterial motility. The swarming and swimming motility dominated by flagella were significantly inhibited (Fig. 2c). Those two showed the movement ability of bacterial population and monomer respectively. As the main movements of bacteria in acute infection, they can help bacteria adhere to the infected site. Therefore, the destruction of flagellum structure will cause the loss of motor ability and reduce the pathogenicity [29]. We also found that the twitching dominated by type IV pili was enhanced, but no statistical difference (Fig. 2c). Some studies have shown that it can help bacteria adhere to the host cells, move on the solid surface through the contraction and extension of pili, causing secondary inflammatory reactions and aggravating the disease [30]. According to the results of the quantitative proteome, we further verified the genes related to flagella function using RT-qPCR and found that the mRNA expression levels of *motA* and *motB* decreased (Fig. 2b). Movement is very important for the survival and pathogenicity of *P. aeruginosa*. Therefore, whether the low motility of *orn* deletion strain will affect the pathogenicity of *P. aeruginosa* will be verified later.

### *Orn* deletion reduced the production of pyocyanin and rhamnolipids in *P. aeruginosa*

**Fig. 3:**
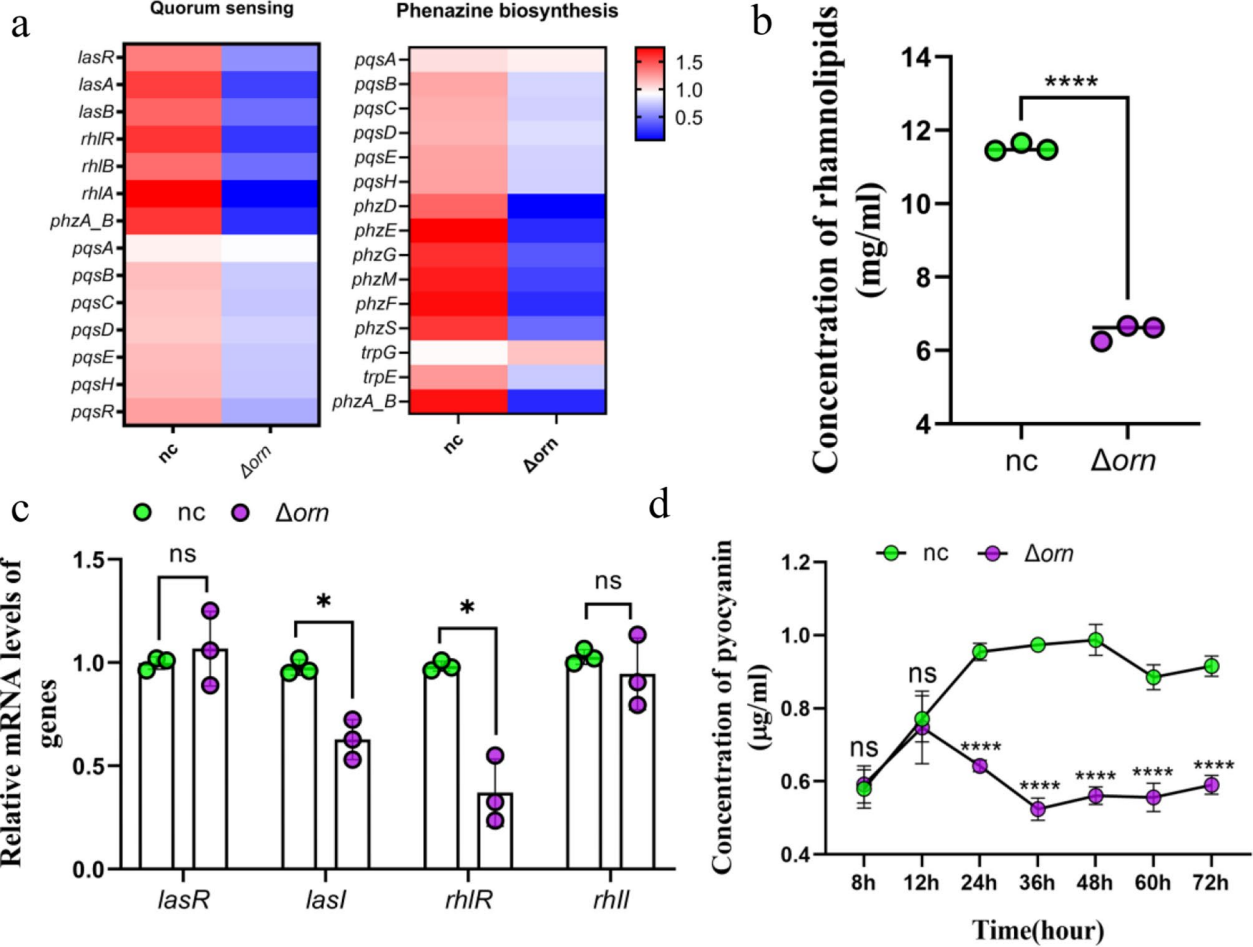
*orn* deletion reduces the production of pyocyanin and rhamnolipids in *P. aeruginosa* (*n* = 4). (**a**) Changes of proteins were associated with the quorum sensing and phenazine biosynthesis of *P. aeruginosa* whithout *orn*. Red boxes represent increased proteins, white boxes represent unchanged proteins and blue boxes represent decreased proteins. The shade of color is positively correlated with the protein expression. (**b**) Rhamnolipids concentration. Significance tested with two-sided student’s *t* test. ****p < 0.0001. (**c**) The relative expression levels of *lasR, lasI, rhlR* and *rhlI* in *P. aeruginosa* were determined by qRT-PCR with *rpoD* as the control housekeeping gene. Significance tested with two-sided student’s *t* test, followed by multiple comparison correction using the Sidak’s test. *p < 0.05. (**d**) Production of pyocyanin in continuous culture within three days. Significance tested with two-sided student’s *t* test, followed by multiple comparison correction using the Sidak’s test. ****p < 0.0001

Then we detected the pyocyanin of *orn* deletion strain. On the quantitative proteome basis, we focused on the expression of related proteins involved in quorum sensing and phenazine synthesis. The expression of the quorum sensing system and phenazine synthesis related proteins of *orn* deficient strains decreased as a whole (Fig. 3a). Our experiments also verified that the quorum sensing system and pyocyanin production capacity of *orn* deletion strains also decreased (Fig. 3c, d). The above results evident that *orn* deletion would lead to the insensitive regulation of *P. aeruginosa* quorum sensing system and reduce the synthesis of pyocyanin.

Furthermore, *rhlR* is the key component of rhlI-rhlR system, which controls the expression of virulence gene of *P. aeruginosa* and directly regulates the synthesis of downstream rhamnolipids. *rhlA* and *rhlB* encode the key enzymes of rhamnolipids synthesis, so the down-regulation of *rhlR* expression will reduce rhamnolipids synthesis (Fig. 3b) and inhibit rhlA-B synthesis (Fig. 3c). The dynamic change of rhamnolipids regulates different metabolic activities of *P. aeruginosa*. For one thing, low concentration of rhamnolipids can enhance the hydrophobicity between cells, which is conducive to bacterial adhesion and promote the formation of micro colonies [31, 32]. For another high concentration, rhamnolipids can be used as an anti-adhesion agent to destroy the formation of biofilm [33].

### *Orn* deletion enhanced the cytotoxicity of bacteria in vitro and in vivo

**Fig. 4:**
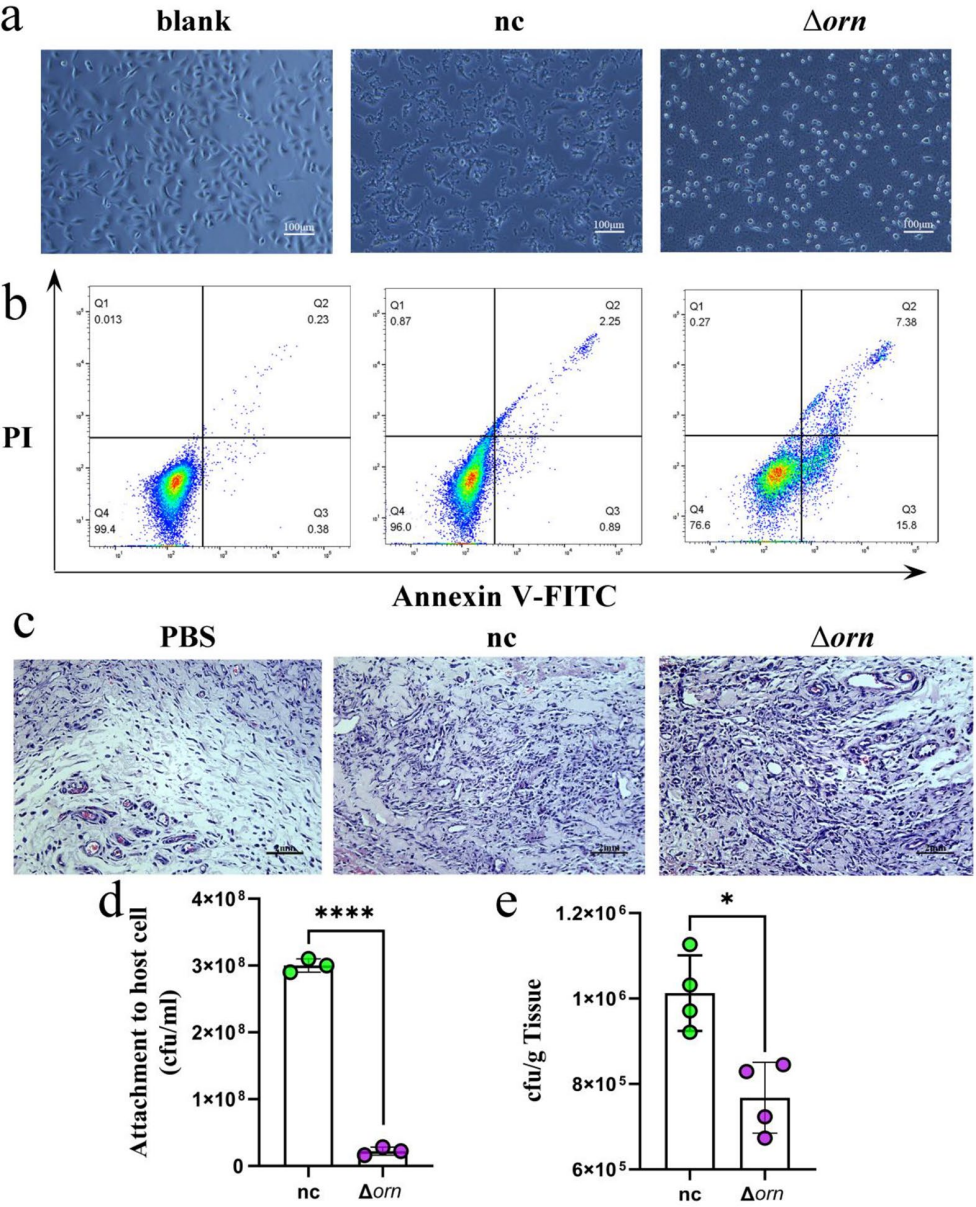
*orn* deletion enhanced *P. aeruginosa* pathogenicity (*n* = 4). (**a**) Apoptosis of A549 cells Blank means add the medium. nc means normal control *P. aeruginosa* ATCC 27853 infected cells. Δorn means *orn* deletion strain infects cells. (**b**) Annexin V/PI double marking A549 apoptosis test. (**c**) After three days of bacterial infection, the wound tissue HE staining. PBS was a blank control. (**d**) Adhesion of bacteria to A549 cells. Significance tested with two-sided student’s *t* test. **** *p* < 0.0001. (**e**) Wound tissue bacterial count. Significance tested with two-sided student’s *t* test. * *p* < 0.05

We unearthed that *orn* deletion will reduce the pyocyanin release and motility of *P. aeruginosa*. However, whether *orn* deletion reduces the overall pathogenicity of *P. aeruginosa* needs further verification. Therefore, we incubated the two strains and their supernatants in A549 cells. The difference of cytotoxicity of supernatant to cells was not obvious (Fig. S3), but the difference of bacterial toxicity to cells was more obvious. Under the microscope, we found that *P. aeruginosa* in the control group adhere to the cells, proliferated and infected. However, the cells co-cultured with *orn* deletion strain were in a severe condition, and most of the cells became round, lost their shape and floated in the culture medium (Fig. 4a), indicating that *orn* deletion will increase the virulence of *P. aeruginosa*. In order to further confirm the cell growth status, we collected the co-cultured cells and detected the apoptosis by flow cytometry. Compared with the control group, the apoptosis caused by *orn* deletion strain was more obvious, indicating that *orn* deletion can enhance the toxicity to A549 cells (Fig. 4b). Besides, we also detected the adherence of bacteria to cells, *orn* deletion reduced the adhesion of *P. aeruginosa* to host cells (Fig. 4d). Proteomic analysis showed that *orn* deletion could increase the protein expression of bacterial protein secretion pathway and lipopolysaccharide synthesis pathway (Fig. S4). In conclusion, *orn* gene enhances the virulence of *P. aeruginosa* to host cells.

We carried out experiments in the rat skin injury model to further verify the regulation of *orn* on the pathogenicity of *P. aeruginosa*. Compared with normal control strain, Δorn strain had more serious inflammatory cell infiltration, that is, more serious bacterial infection (Fig. 4c). After three days of infection, the wound tissue was taken out, and the number of bacteria in Δorn infected wound was significantly reduced compared with normal control group (Fig. 4e). The above experiments confirmed that *orn* deletion could increase the pathogenicity of *P. aeruginosa* in rat skin wound model.

### *Orn* had different regulatory effects on c-di-GMP under different culture conditions

**Fig. 5:**
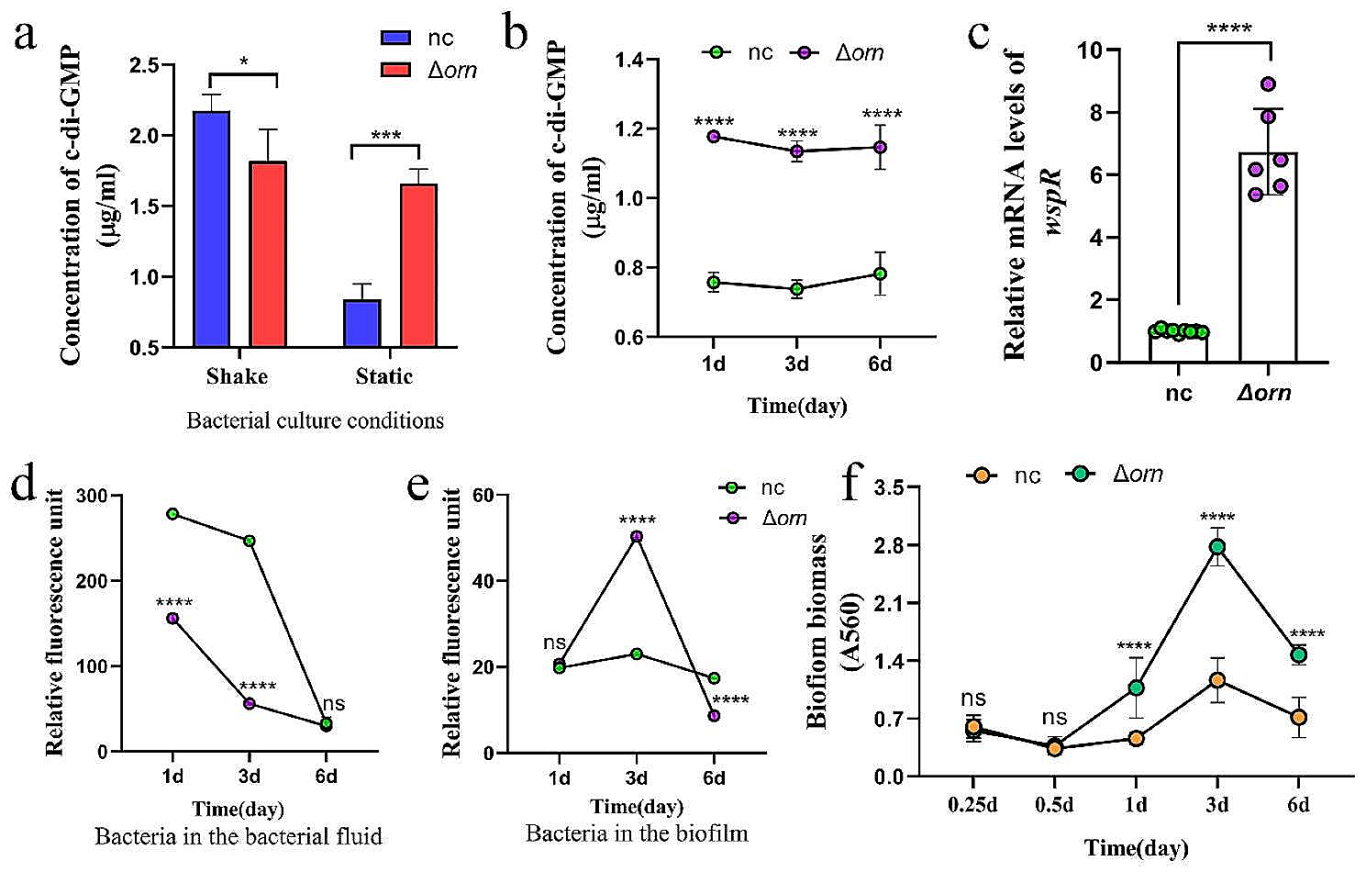
The regulatory effects of *orn* on c-di-GMP were dynamic (*n* = 4). (**a**) Concentration of c-di-GMP under different culture conditions, *n* = 3, Significance tested with two-sided student’s *t* test. *** *p* < 0.001. (**b**) Changes of c-di-GMP concentration during biofilm formation, 1 d means initiation of biofilm formation. 3 d means microcolony formation stage. 6 d means the biofilm maturation stage. Significance tested with two-sided student’s *t* test, followed by multiple comparison correction using the Sidak’s test. **** *p* < 0.0001. (**c**) The relative expression levels of *wspR* in *P. aeruginosa* was determined by qRT-PCR with *rpoD* as the control housekeeping gene. Significance tested with two-sided student’s *t* test. **** *p* < 0.0001. (**d, e**) Bacterial activity in bacterial fluid or biofilm. ns means no difference. **** *p* < 0.0001. (**f**) Biofilm biomass tested by crystal violet assay. Significance tested with two-sided student’s *t* test, followed by multiple comparison correction using the Sidak’s test. **** *p* < 0.0001

C-di-GMP, an important second messenger in bacteria, can regulate bacterial movement, the production of virulence factors and extracellular polysaccharides, as well as the formation of *P. aeruginosa* biofilm [34]. *Orn* affects the synthesis and metabolism of c-di-GMP by degrading pGpG because the accumulation of pGpG will lead to the accumulation of c-di-GMP. When verifying the effect of *orn* on c-di-GMP, interestingly, we found that *orn* deficient strains showed different c-di-GMP levels under different culture conditions. During shaking culture, the concentration of c-di-GMP in Δorn group was lower than that of the normal control group (Fig. 5a), and the expression of c-di-GMP synthase, diguanylate cyclase *wspR* increased (Fig. 5c), proteomics also showed high levels of WspR. In contrast, when the bacterial suspension was cultured statically, the concentration of c-di-GMP in the bacterial suspension of normal control group decreased significantly, and the concentration of c-di-GMP in the bacterial suspension of Δorn group had no significant difference in the two culture states, and was higher than that of normal control group (Fig. 5a).We think that, in the static culture stage, the outcome of *orn* deletion is not so much to promote the synthesis of c-di-GMP as to maintain the level of c-di-GMP, so as to promote the formation of biofilm.The two culture states reflected the different life styles of bacteria, namely planktonic life and sessile life. Biofilm is the main way of fixed life of *P. aeruginosa*. Therefore, we also detected the changes of c-di-GMP level in different stages of biofilm formation and found *orn* deficient strains can maintain a high level of c-di-GMP production during biofilm formation (Fig. 5b), showing enhanced biofilm formation ability (Fig. 5f). At the same time, the bacterial vitality also changes due to different culture conditions. In the floating state, the activity of *P. aeruginosa* in *orn* deletion group was significantly lower than that in normal control group (Fig. 5d). However, in the biofilm formation stage, the bacterial activity of Δorn group changed significantly. In the micro colony formation stage, about the third day of culture, the bacterial activity increased significantly, indicating that there were vigorous biological metabolic activities in the biofilm. By the sixth day of culture, the bacterial activity of Δorn group decreased, which may promote the bacteria to break the biofilm and spread out in advance in order to survive(Fig. 5e). Biofilm formation is also one of the main reasons why it is difficult to completely remove *P. aeruginosa*. High biofilm forming ability always means that it is easier to develop into multi-drug resistant bacteria. Therefore, *orn* may have clinical significance for the formation of *P. aeruginosa* biofilm. The above experiments showed that *orn* deletion can promote the synthesis of c-di-GMP and further promote the formation of *P. aeruginosa* biofilm.

### *Orn* deletion promoted the aggregation of *P. aeruginosa*

**Fig. 6:**
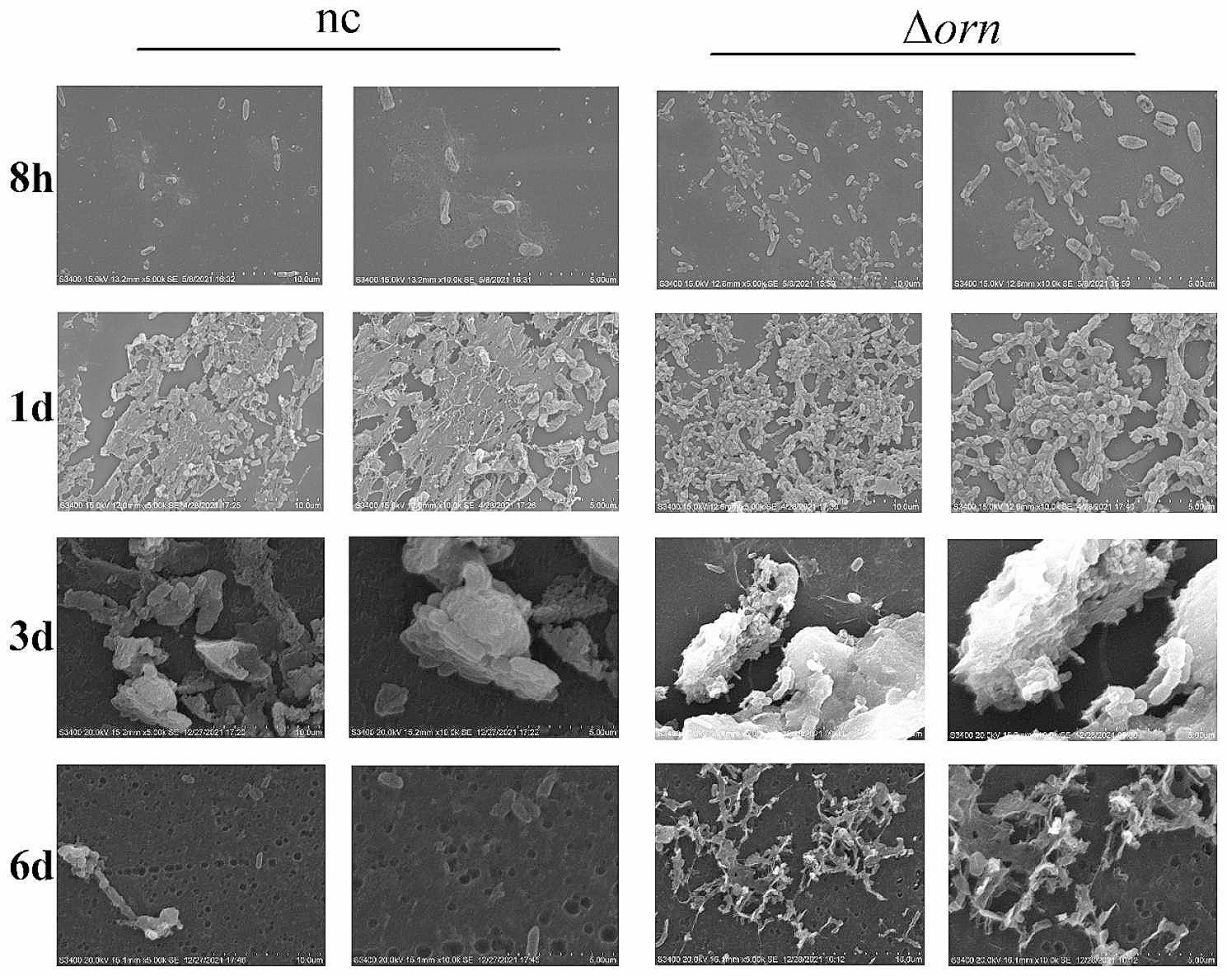
*orn* deletion enhanced *P. aeruginosa* aggregation. Colony status of *P. aeruginosa* after static culture for 8 h to 6 days with magnifications of 5.00 and 10.00 K, respectively

Scanning electron microscopy was used to detect the aggregation and biofilm formation ability of *orn* deficient *P. aeruginosa*. Round overslip were pre-placed in the six well plate, static culture was added after each bacterial suspension was added, and the colonies were taken out after 8 h to 6 days of culture. Obviously, *orn* deletion increased the aggregation ability of *P. aeruginosa* (Fig. 6). Static culture for 8 h to one day is the initial formation stage of *P. aeruginosa* biofilm. There are a few bacteria in the normal control group, which gather in a single layer, while in Δorn group, more bacteria accumulated on the round overslip and tended to stack. The third day of culture is the stage of biofilm microcolony formation, and the bacteria begin to proliferate. Both groups had a bacterial stacking phenomenon. The membrane complex composed of extracellular polysaccharide and protein wrapped the colony, and *orn* deletion leads to larger micro colony formation and thicker cell membrane complex formation. Finally, on the sixth day of culture, the biofilm is mature. The membrane covering the colony begins to separate, and the bacteria become free and start the next cycle. There was almost no bacterial residue on the round overslip in the nc group, and the footprints and grooves of the lowest bacterial attachment can be seen. Interestingly, Δorn group still has a large number of bacterial residues, meanwhile the speed of biofilm dispersion and separation becomes slower. It can be considered that *orn* deficiency promotes the formation of biofilm and slows down the rate of biofilm detachment. Therefore, we believe that *orn* can regulate the aggregation and biofilm formation of *P. aeruginosa*, which brings great challenges to the clinical treatment of *P. aeruginosa* infection.

## Discussion

*P. aeruginosa* has a variety of virulence factors and sensitive environmental adaptability mechanisms, which constitute the main pathogenic factors. Studies have shown that when the living environment is nutrient-poor, *P. aeruginosa* can reassemble its own metabolic reorganization and produce new metabolic pathways to survive by using the metabolites of the host [35]. *P. aeruginosa* regulates the expression of related genes and intercellular signal pathways through complex signal system networks, mainly quorum sensing system and bacterial chemotaxis. Quorum sensing system is divided into LasI/LasR system, which regulates the expression of virulence factors such as elastase, proteolytic enzyme and exotoxin; RhlR system regulates the expression of rhamnolipids; Quinolone system regulates the expression of pyocyanin. We found that *orn* deficient strains showed decreased expression of quorum sensing system regulatory proteins, resulting in dropped synthesis of pyocyanin and rhamnolipids. Compared with normal control, the concentration of *orn* deletion strain c-di-GMP changed greatly, and the production of sessile life was about twice that of planktonic life. Surely, this is also accorded with the regulation process of c-di-GMP. High concentration of c-di-GMP is mostly manifested in the period of chronic infection, resulting in the weakening of bacterial motility and pathogenicity, and the formation of biofilm to resist the clearance of the host immune system. On the other hand, low concentration of c-di-GMP will enhance the motility and pathogenicity of bacteria, which makes the bacteria develop into the acute infection stage, showing the flexible regulation of environmental adaptability of *P. aeruginosa*.

The key points of bacteria causing host pathogenicity are the contact between bacteria and host and the existence of specific receptors on host cells. As the first line of defense of human immunity, skin and mucosa are very important for pathogenic bacteria to start infection. *orn* deletion will lead to flagellum dysfunction of *P. aeruginosa* and weaken the motility and adhesion of bacteria, which is unfavorable to bacterial pathogenicity, but on the other hand, the weak motility of bacteria conduce to the formation of biofilm [36]. In the process of biofilm formation of *P. aeruginosa*, the movement of bacterial flagella is dynamic. In the initial stage, flagella is inhibited, which is conducive to bacterial aggregation and micro colony formation. In the later stage, when biofilm matures, flagella inhibition is relieved, resulting in biofilm rupture and bacterial diffusion [37].

Pyocyanin is an antibiotic compound with broad-spectrum antibacterial activity, which is released with the cleavage of *P. aeruginosa*. It not only acts to kill other bacteria, but also attacks the host immune system by inducing neutrophil apoptosis [38]. It is found that pyocyanin is also involved in a variety of important bioactive processes as a signal molecule of quorum sensing PQS [39]. At present, some studies have shown that the expression level of genes related to pyocyanin synthesis in *orn* mutant strain is significantly increased through RNA-seq results. However, from the perspective of quantitative proteomics, we have come to different conclusions. KEGG pathway analysis shows that the overall expression of phenazine synthesis pathway is down-regulated, especially pyocyanin synthesis regulates *phzA/B, phzG* and *phzS* were down-regulated significantly, and pyocyanin production was down-regulated in *orn* deficient strains. We suspect that it may be due to the different bacterial culture states. We tested the synthesis ability of pyocyanin in shaken culture and static culture and found that *orn* deletion does weaken the synthesis ability of pyocyanin.

Data show that microorganisms related to biofilm colonize a variety of medical devices, and get involved in more than 80% of chronic inflammation and infectious diseases [6]. We found the enhancement of biofilm formation ability of *orn* deletion strains. In Δorn strain, the content of polysaccharide in the biofilm component did not increase significantly. Bacterial biofilms are also divided into mucinous and nonmucinous types. Mucinous biofilms are mostly composed of polysaccharides, while non-mucinous biofilms are mostly composed of proteins. We speculate that *orn* may regulate the protein components of biofilms. Moreover, *orn* deletion will lead to the accumulation of bacterial c-di-GMP and the disorder of *P. aeruginosa* quorum sensing system. Therefore, we believe that the disordered quorum regulation system may lead to the imbalance of aggregation ability, and finally lead to the enhanced, but structure-special biofilm formation.Unfortunately, the specific regulatory mechanism has not been clarified in this study. In addition, although studies have confirmed that *orn* can affect the formation of biofilm through c-di-GMP, in these proteomic results, we also found a significantly increased signal molecule ppGpp, which mainly mediates the production of bacterial stress response. Relevant studies have shown that the metabolic level of bacterial ppGpp regulates the expression of virulence factors and bacterial motility, and can also promote the formation of bacterial biofilm [40]. Therefore, we suspect that *orn* regulated biofilm formation may not only be regulated by c-di-GMP alone, but also by other signal molecules, which may be the main content of our subsequent study.

## Conclusion

Based on our findings, it is established that *orn’s* metabolic regulation of *P. aeruginosa* is global. *orn* deletion can reduce the energy metabolism, adversely affect the quorum sensing system, two-component system and bacterial chemotaxis, and weaken the bacterial motility. In terms of pathogenicity, *orn* deletion can result in a decrease in pyocyanin and rhamnolipids. However, it increases bacterial protein secretion, especially in the type III and VI secretion system. The lipopolysaccharide synthesis pathway was up-regulated and the cytotoxicity in vitro and in vivo was increased. In addition, *orn* deficiency can promote the level of c-di-GMP and the formation of biofilms of *P. aeruginosa*. *orn* can significantly affect the metabolism and regulate the global transcription of bacteria, which may explain the high adaptability, high toxicity and strong drug resistance of *P. aeruginosa*.

### Electronic supplementary material

Below is the link to the electronic supplementary material.

**Supplementary Material 1:** Supplementary Figure S1-S5. Supplementary Table S1-S2

## Data Availability

All the data used found in during the experiments will be supplemented upon request.
