# Supplementary material for: Oligoribonuclease mediates high adaptability of P. aeruginosa through metabolic conversion
Source: BMC Microbiol. 2024 Jan 19;24:25. doi: 10.1186/s12866-023-03175-3 (PMC10797966; doi:10.1186/s12866-023-03175-3)
Supplement: Supplementary file 1 — Supplementary Material 1: Supplementary Figure S1-S5. Supplementary Table S1-S2 [file 12866_2023_3175_MOESM1_ESM.docx]

**Supplementary materials**


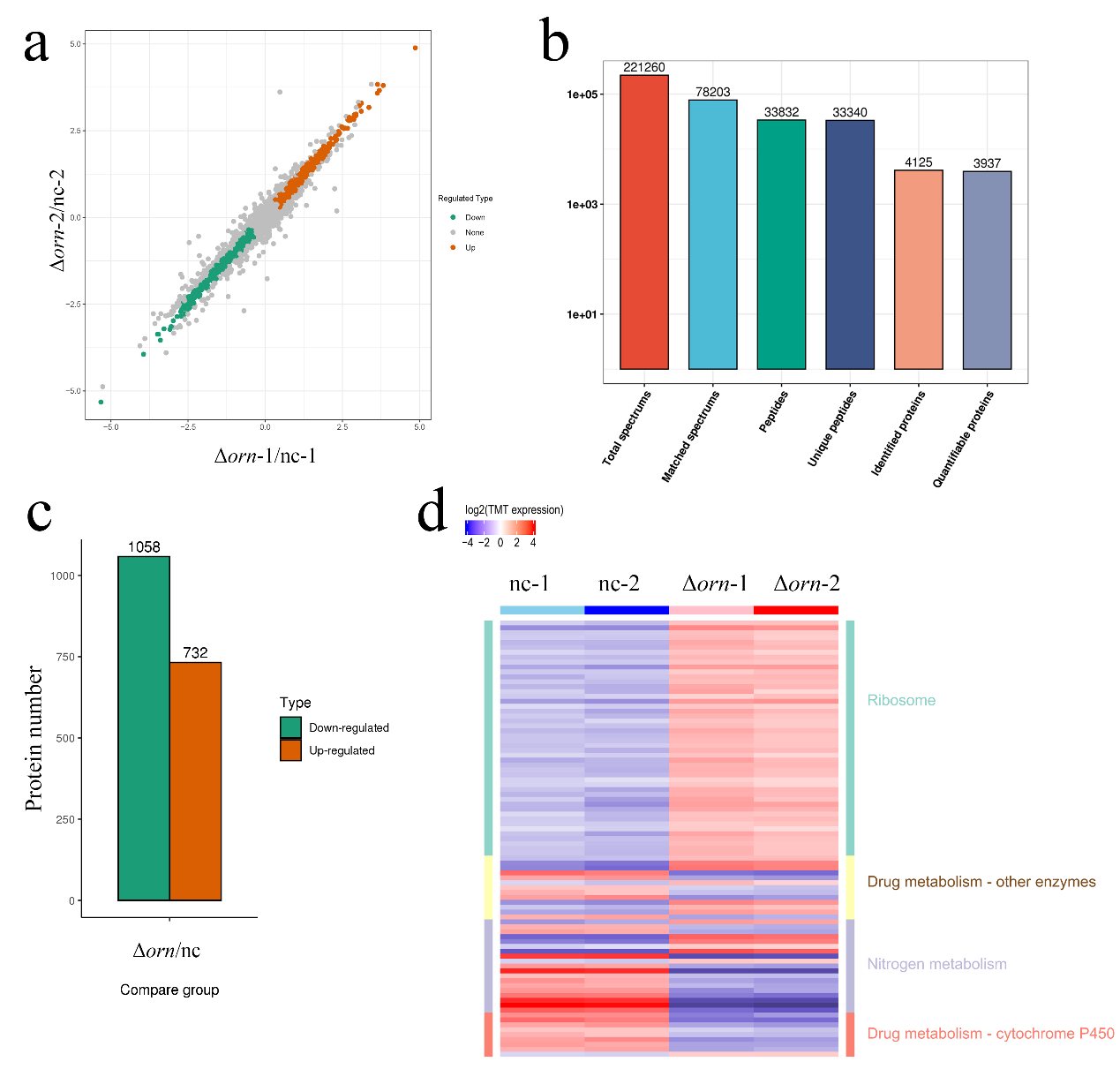
Figure S1. Identification of differentially expressed proteins in *orn* deficient *P. aeruginosa* by TMT labeled quantitative proteomics. (a) The scatter plot map of differentially expressed proteins. (b) MS/MS spectrum database search analysis summary. (c) The number of differentially expressed proteins identified by TMT-labeled quantitative proteomics. (d) The quantitative value heat map of differential proteins corresponding to the first four pathways of KEGG was significantly enriched.


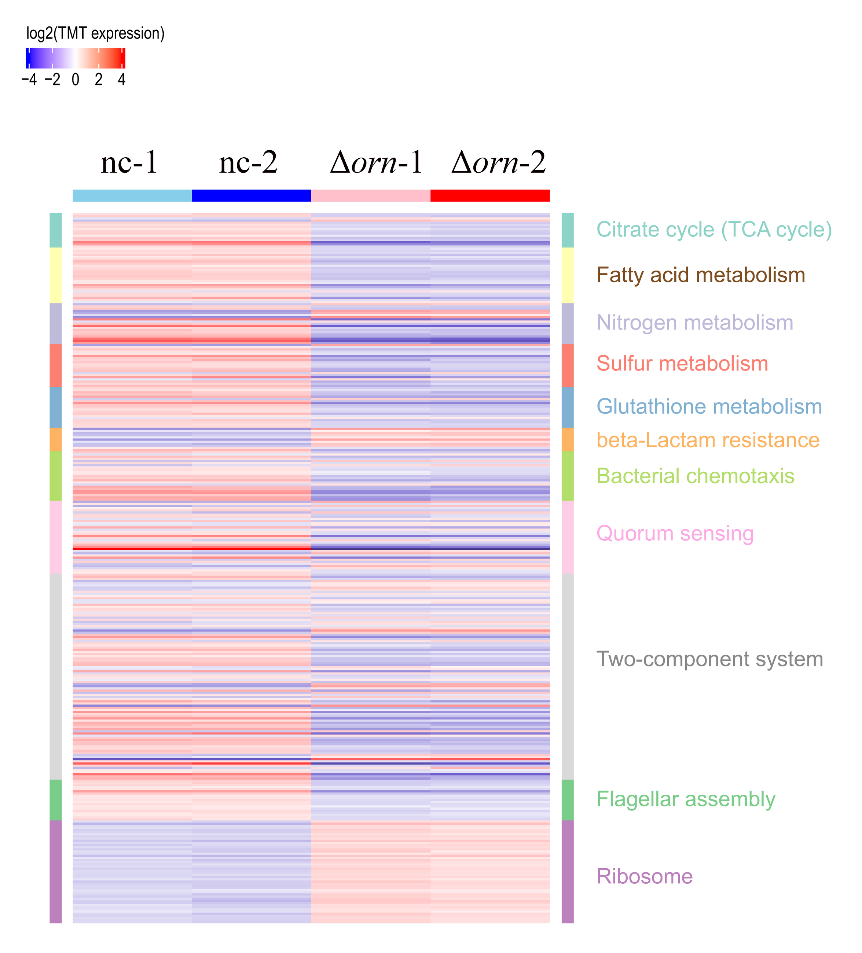


Figure S2. KEGG pathway clustering heat map of the selected pathways proteins. The deeper the blue color, the more significant the enrichment is.


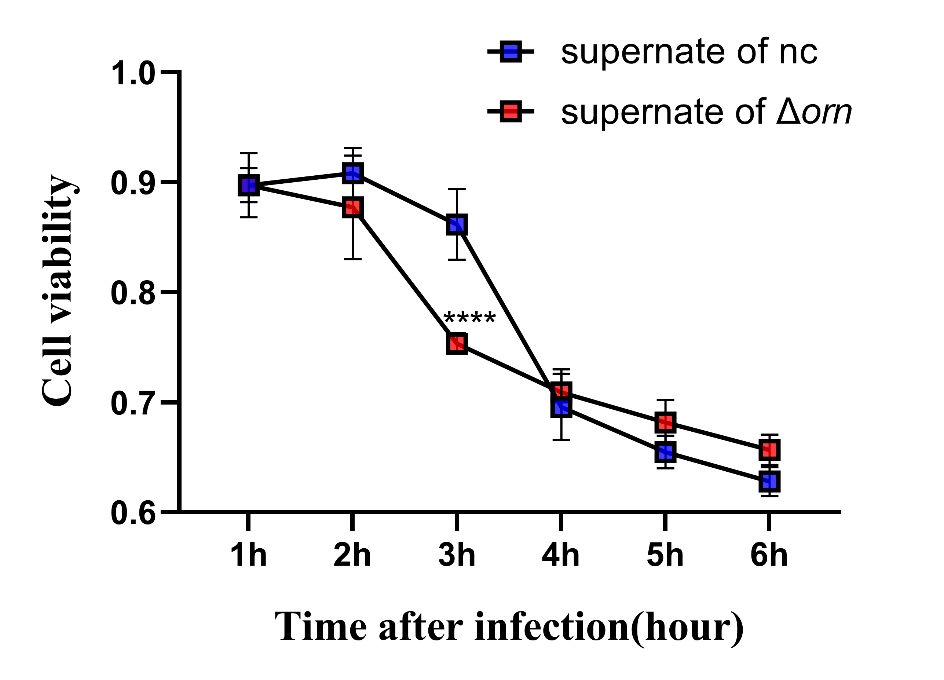


Figure S3. Bacterial supernatant-infected A549 cells. Dilute the bacterial suspension until OD600 equal to 1, centrifuge and collect the supernatant, and then add the cell culture medium. Dots represent the means and SDs of five biological replicate. Significance tested with two-sided student’s *t* test, followed by multiple comparison correction using the Sidak’s test. **** *p* < 0.0001.


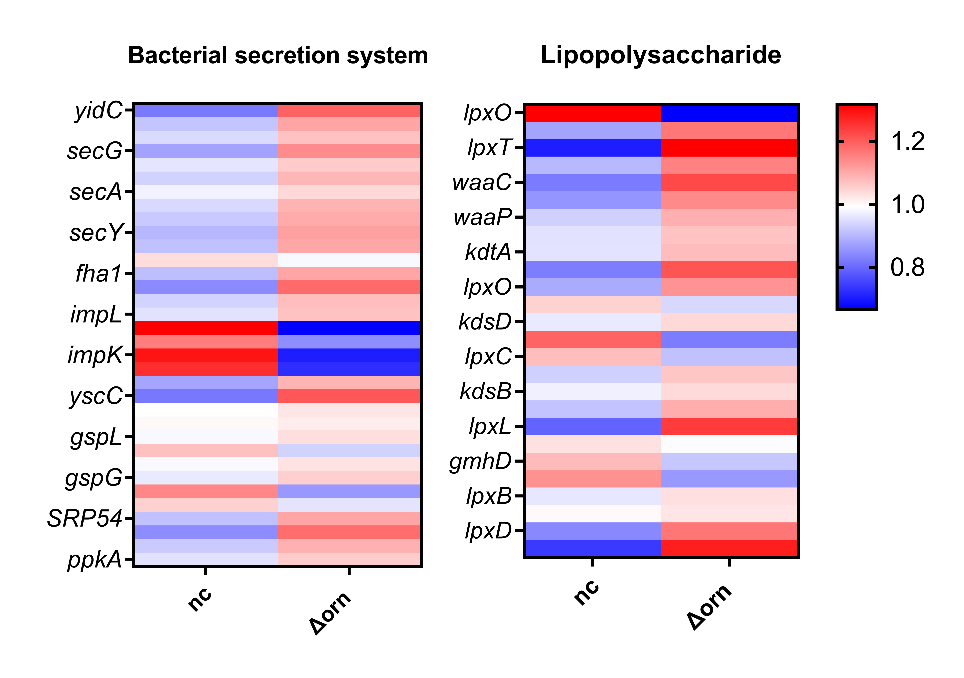


Figure S4. Changes of proteins associated with the bacterial secretion and lipopolysaccharide of *P. aeruginosa*. Red boxes represent increased proteins, white boxes represent unchanged proteins and blue boxes represent down-regulated proteins. The shade of color is positively correlated with the protein expression.


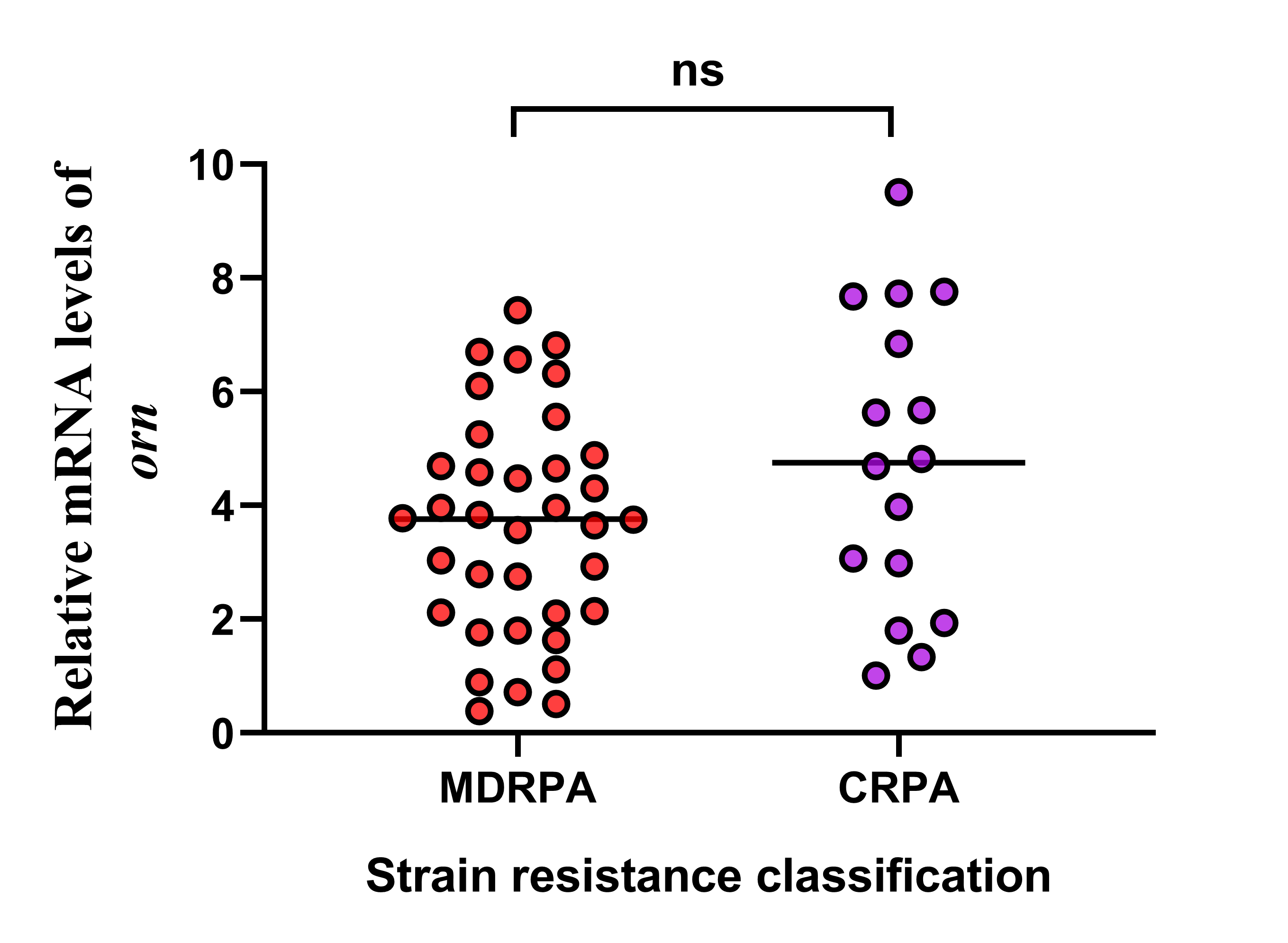


Figure S5. Clinical orn expression. MDRPA means multiple drug resistance of *P. aeruginosa* isolated from clinic. CRPA means carbapenem resistance of *P. aeruginosa* isolated from clinic. Significance tested with two-sided student’s t test.

| **Table S1.Bacterial strains and cells used in the study** | | |
| --- | --- | --- |
| **Strains** | **Description** | **Source** |
| *P.aeruginosa* ATCC 27853 | Wild type. | Lab stock |
| Clinical strains of *P.aeruginosa* | Carbapenems were resistant, of which 36 strains were multidrug resistant. | Hunan Brain Hospital |
| *E.coli* DH5αλpir | Amplification of suicide plasmid (targeting plasmid) pCVD442-Δ*orn*::Gm. | Sangon Biotech,China |
| *E.coli* β2155 | The donor strain used for the conjugation experiment contains the targeting plasmid pCVD442-Δ*orn*::Gm. | Sangon Biotech,China |
| *P.aeruginosa* ATCC27853/pCVD442-Δ*orn*::Gm | Its genome was inserted into the target plasmid. | This study |
| *P.aeruginosa* ATCC27853/Δ*orn*::Gm | The *orn* gene was replaced by gentamicin resistance gene. | This study |
| **Cells** | | |
| A549 | Lung cancer human alveolar basal epithelial cells | Lab stock |
| **Plasmids** | | |
| pJQ200SK | Contains gentamicin resistance gene. | Sangon Biotech,China |
| pCVD442 | Suicide plasmid for *orn* knockout. | Sangon Biotech,China |
| pCVD442-Δ*orn*::Gm | Insert gene targeting fragment: upstream homologous arm - gentamicin resistance gene - downstream homologous arm. | This study |

| **Table S2.Primers used in this study** | | |
| --- | --- | --- |
| **Primers** | **Sequence (5'-3')** | **Function** |
| *orn*-5F | GACTACCACAAGCTGATCCGCAAG | Cloning of upstream homologous recombination. arm fragment of *lrn*. |
| *orn*-5R | TTCATCAAGCTCTGATCCGTCCGG |  |
| *orn*-3F | GTCGCTATGCCGTGCATTCTAGC | Cloning of downstream. homologous recombination arm fragment of *lrn*. |
| *orn*-3R | GACGAAGACGCTCACGTGACC |  |
| *orn-Gm*F | CCGGACGGATCAGAGCTTGATGAATTGTGACAATTTACCGAACAAC | Cloning of gentamicin (*Gm*) resistance gene. |
| *orn-Gm*R | CACGCTAGAATGCACGGCATAGCGACAGAAATGCCTCGACTTCGC |  |
| *wspR*-F | GCGGTCATGGTACTGCTTGTCG | RT-qPCR |
| *wspR*-R | TGCGGATCGGAACAGAAATGGAAG |  |
| *lasR*-F | GCTGGAACGCTCAAGTGGAAAATTG | RT-qPCR |
| *lasR*-R | TTCTCGTAGTCCTGGCTGTCCTTAG |  |
| *lasI*-F | TGCGTGCTCAAGTGTTCAAGGAG | RT-qPCR |
| *lasI*-R | ATAAGGACTGAGTGCGTCATAACCATC |  |
| *rhlI*-F | CTCTGAATCGCTGGAAGGGCTTTC | RT-qPCR |
| *rhlI*-R | TTTGCGGATGGTCGAACTGGTC |  |
| *rhlR*-F | GAGCGATACCAGATGCAGAACTACG | RT-qPCR |
| *rhlR*-R | TCCAGACCACCATTTCCGAGGAG |  |
| *motA*-F | GTGCTGAAGGATGAGCGGATGAC | RT-qPCR |
| *motA*-R | ATGCTCCAGGTCCTCCTTGAGAC |  |
